# Prevalence of Antenatal Depression and Associated Factors among Pregnant Women Attending Antenatal Care at Dubti Hospital: A Case of Pastoralist Region in Northeast Ethiopia

**DOI:** 10.1155/2018/1659089

**Published:** 2018-10-02

**Authors:** Yihalem Abebe Belay, Nurilign Abebe Moges, Fetuma Feyera Hiksa, Kassahun Ketema Arado, Misgan Legesse Liben

**Affiliations:** ^1^Department of Public Health, College of Health Science, Debre Markos University, Debre Markos, Ethiopia; ^2^Department of Nursing, College of Health Science, Debre Markos University, Debre Markos, Ethiopia; ^3^Department of Public Health, Faculty of Health Sciences, Woldia University, Amhara, Ethiopia

## Abstract

**Background:**

Globally, depression affects an estimated 10 % to 20% of women during pregnancy. There is limited evidence on antenatal depression in Northeast Ethiopia. This study aimed to assess prevalence of antenatal depression and associated factors among Dubti Hospital Antenatal care attendants.

**Methods:**

Institution based cross-sectional study was conducted among 363 Antenatal care attendants at Dubti Hospital from March 07 to May 07, 2016. Beck's Depression Inventory tool was used to collect data. Data were entered into Epi-Data 3.1 and analyzed using SPSS 20. Bivariable and multivariable logistic regression analyses were fitted. Variables having *p value* < 0.05 were considered as statistically significant.

**Results:**

A total of 357 pregnant women were interviewed. The prevalence of antenatal depression was 17.9% [95% CI (14.0, 22.0%). Pregnancy planning [AOR: 0.04; 95% CI (0.014, 0.114), social support [AOR: 0.21; 95% CI (0.07, 0.66), and marital conflict [AOR: 6.45; 95% CI (2.1, 17.9)] were significantly associated with antenatal depression.

**Conclusions:**

Nearly one in five pregnant women had depression. Marital conflict, pregnancy planning, and social support were significant predictors of antenatal depression. Dubti Hospital should strengthen its effort on prevention of unplanned pregnancy. Healthcare workers in antenatal care unit have to deal with marital conflict and social support as part of their routine investigation to avoid complications through early detection of antenatal depression.

## 1. Introduction

Antenatal depression is defined as the occurrence of a depressive episode in women during pregnancy. The World Health Organization (WHO) ranked depression as a single largest contributor to global disability in 2015. Depression has been reported more common among female population group as compared to male population group [1]. Depression affects an estimated 10 % to 20% of pregnant women worldwide. Depression is more prevalent among women in low- and middle-income countries compared to those women in high income countries [2]. The prevalence of antenatal depression varies across different parts of the world. It has been reported to be 47% in rural South Africa [3], 39.5% in Tanzania [4], 28.5% in China [5], 14.9 % in Italy [6], 13.8% in Sabah Malaysia[7], 13.2 % in Germany [8], and 10.9% in Turkey [9]. In Ethiopia, the prevalence of antenatal depression is reported to be 31.2% in Adama Hospital [10], 31.1 % in Maichew [11], 29.5 % in Sodo district of Gurage Zone [12], 24.9% in Addis Ababa Public Health Centers [13], 23% in Gondar University Hospital [14], and 11.8% in Debretabor Town [15].

Antenatal depression is a significant predictor for postnatal depression [16–22]. Beyond the woman, it is also an independent risk factor for offspring depression up to age of 18 [23]. Antenatal depression is associated with operative delivery and preeclampsia [24], preterm birth [25, 26], and low-birth weight [25]. But, it is not associated with pregnancy loss or infant death [12]. Women with depressive symptoms had an increased risk of having more nonscheduled ANC visits and increased number of emergency healthcare visits for pregnancy-related emergencies [12]. Increased depressive symptoms are also associated with no engagement in favorable health practices during pregnancy [27]. A cohort study in Malaysia revealed that women with antenatal depression were more likely to stop breast feeding before six months than their counterparts [28].

Previous studies have indicated that some of the most common risk factors for antenatal depression include younger age, low income, unemployment, single marital status, low educational status, psychiatric histories, use of substances, lack of social support, marital conflict, multigravidity, less number of parities, more number of children, unplanned pregnancy, history of abortion, and a history of obstetric complications [2, 3, 5, 6, 10, 11, 13, 15, 29–33]. The Government of Ethiopia has launched and enforced a mental health strategy (2012/13-2015/2016) which aimed to provide mental health services at all levels of the existing health system including health posts. However, still there is a gap in mainstreaming a mental health service with the routine maternal health services, like antenatal care in the country. Previous studies have been conducted in Central and Northwest Ethiopia reporting different findings about antenatal depression. There is limited evidence regarding the prevalence and associated factors of antenatal depression in Northeast part of Ethiopia. Therefore, this study aimed to assess the prevalence and associated factors of depression among pregnant women attending antenatal care at Dubti Hospital in Afar Regional State, Northeast Ethiopia.

## 2. Materials and Methods

### 2.1. Study Design, Area, and Period

An institution based cross-sectional study was conducted at Dubti Hospital. The hospital is located 10 km far from Samara, the capital city of Afar National Regional State. It is one of the six hospitals in the region, which offers a full range of healthcare services including antenatal care and mental health services. Apart from other services, the hospital provides a routine antenatal follow- up care for pregnant women. An evidence reported from the hospital showed that a total of 4560 pregnant women receive antenatal care annually from this hospital [34]. The study was conducted from March 07 to May 07, 2016.

### 2.2. Sample Size Determination and Sampling Procedure

A sample size of 363 was calculated using a single population proportion formula:

(1) $$\begin{array}{c} n=\frac{\left(za/2\right)2p\left(1-p\right)}{d2} \end{array}$$

*Assumptions*. n is required sample size, Z is critical value for normal distribution at 95% confidence level (1.96), d = 0.05 (5% margin of error), P=31.2% (proportion of pregnant women having antenatal depression) [10], and an estimated nonresponse rate is 10%.

First, pregnant women at any trimester of pregnancy who were attending antenatal checkup at Dubti Hospital during the study period were included. Then, systematic random sampling technique was used to select every other pregnant woman. Pregnant women who were seriously ill and unable to hear and/or speak were excluded from the study.

### 2.3. Data Collection Process and Instrument

Data were collected using a pretested-interviewer administered structured questionnaire. The questionnaire was prepared first in English and translated into Amharic and then back to English to check for its consistency. The Amharic version of the questionnaire was used to collect the data.

Two female diploma nurses and one BSc public health professional were recruited as data collectors and supervisor, respectively. The data collectors and supervisor were trained for two days on the study objective and data collection process. The questionnaire was pretested on 5% of the sample size at Aysaita Hospital, and amendments on the questionnaire were made accordingly. Intensive supervision was done by the supervisor and principal investigator throughout the data collection period.

### 2.4. Study Variables

The dependent variable in this study was antenatal depression. Beck's Depression Inventory Version-II (BDI-II) was administered to detect depression.

BDI is a reliable and valid measure of depression in a range of populations in most of the countries in the world including Ethiopia. It consists of 21 items, and each of the items describes a specific symptom of depression. Each statement is scored on a 4-point scale (0 to 3) and a total score is obtained by summing the ratings for each statement. Therefore, the total score ranges from 0 to 63 [35]. Then, a score of 17 and above was used as a cutoff point to detect antenatal depression in this study [7, 9]. Finally, pregnant women who scored 17 and above were coded as “1” and those who scored less than 17 were coded as “0” for regression analysis.

The independent variables were socioeconomic characteristics (maternal age, educational status, marital status, occupation, and average family monthly income), obstetric factors (gravidity, parity, number of children, history of abortion, history of stillbirth, history of pregnancy complication, and pregnancy planning), psychosocial factors (social support and relationship with partner), history of a depressive disorder (in the women and family), and substance use. Average family monthly income was defined based on minimum Ethiopian monthly wage of 21 USD [36] that was about 500 Ethiopian birr during the study period.

In this study, ever use of substance was defined as pregnant women who had used a psychoactive substance at least once in their lifetime and using psychoactive substance within 30 days preceding the study as current use of substance.

Social support was measured using the Maternity Social Support Scale (MSSS) developed by Webster and colleagues [37]. The scale contains six items and includes questions on family support, friendship network, help from spouse, conflict with spouse, feeling controlled by spouse, and feeling unloved by spouse. Each item was measured on a five-point Likert scale and a total score of 30 was possible. Social support was classified into three categories: high social support (for scores 24–30), medium social support (18–23), and low social support (below 18) categories.

### 2.5. Data Processing and Analysis

The data were checked for completeness and consistencies. Data were also cleaned, coded, and entered into Epi-Data software version 3.1 and then exported to SPSS version 20 statistical package for analysis. The crude odds ratios with 95% confidence interval were estimated in the binary logistic regression analysis to assess the association between each independent variable and the outcome variable. Variables with *p* value <0.25 in the bivariable logistic regression analysis were considered in the multivariable logistic regression analysis. The Hosmer-Lemeshow goodness-of-fit with enter procedure was used to test for model fitness. Adjusted odds ratios with 95% confidence interval were estimated to assess the strength of the association, and variables with *p* value <0.05 were considered significant factors.

## 3. Results

### 3.1. Sociodemographic Characteristics of the Study Participants

A total of 357 pregnant women were included in the study, resulting in a response rate of 98.3%.

The mean (±SD) age of participants was 25.97 (±5.61) years with a range of 16 years to 43 years. Three hundred forty-five (96.6%) and 205 (57.4%) women were married and had attended formal education, respectively. Almost 90 percent of study participants had reported that they earn an average family monthly income of more than 500 Ethiopian Birr (the minimum Ethiopian wage during that time) (Table 1).

### 3.2. Obstetric Characteristics

More than three-fourth (76.2%) of pregnant women were in either the second or third trimester during the time of the study. Two hundred eighty-four (79.6%) of the women had planned their current pregnancy. Of 221 women who had a history of pregnancy, 55(24.9%) had a history of complication during previous pregnancy (Table 2).

### 3.3. Psychiatric History and Psychosocial Support

About 97 (27.2%) of the study participants reported a previous history of depression. The same proportion of mothers (27.2%) had encountered a conflict with their husbands in the last 12 months preceding the study (Table 3).

### 3.4. Psychoactive Substance Use

Twenty-one (5.9%) of the study participants had used alcohol at least once in the last 30 days. None of the participants had reported current khat chewing. None of the study subjects had used tobacco and shisha in their life time (Table 4).

### 3.5. Factors Associated with Antenatal Depression

The prevalence of antenatal depression was 17.9% (95% CI: 14.0 – 22.0%). Bivariable logistic regression analysis showed that maternal education, average family monthly income, history of complication in previous pregnancy, pregnancy planning, social support, marital conflict, and previous history of depression were statistically associated with antenatal depression at *p* value <0.05 (Table 5).

In multivariable logistic regression analysis marital conflict, pregnancy planning, and social support were found to be significantly associated with antenatal depression at *p* value <0.05 (Table 5).

Those women who had marital conflict were about six times more likely to have antenatal depression as compared to those who had no marital conflict [AOR=6.45(2.1, 17.9)]. Women who had planned their current pregnancy [AOR=0.04(0.01, 0.11)] were 96% less likely to have antenatal depression as compared to women who had no planned pregnancy. Compared to women who had low social support, women who had medium social support [AOR=0.21 (0.07, 0.66)] were 79 % less likely to have antenatal depression.

## 4. Discussion

Mental well-being is a fundamental component of WHO's definition of health. Good mental health enables people to realize their potential, cope with the normal stresses of life, work productively, and contribute to their communities. Depressive disorder is an important health problem globally. This study examined the prevalence of antenatal depression among pregnant women attending antenatal care service in Dubti Hospital and explored its associated factors. A high prevalence of antenatal depression was found in this study. About 17.9% of pregnant women who had antenatal care follow -up at Dubti Hospital scored 17 and above in Beck's Depression Inventory (BDI-II) tool. A relatively similar prevalence was reported from India (16.3%) [31] and Ethiopia (19.9%) [38]. The prevalence of antenatal depression in this study was lower than the findings from rural South Africa (47%) [3], Tanzania (39.5%) [4], Adama Hospital (31.2%) [10], Addis Ababa Public Health Centers (24.9%) [13], and Gondar University Hospital (23%) [14]. However, it was higher than the finding in Debretabor town (11.8%) [15]. This could be due to the sociodemographic and economic differences. The geographic and cultural variations might also attribute such differences among these studies. In addition, the measurements used to ascertain the outcome variable might differ. Beck's Depression Inventory (BDI), Edinburgh Postnatal Depression Scale (EPDS), and Patient Health Questionnaire (PHQ) were used in these studies which might give a varied estimate of the prevalence of antenatal depression in such studies.

With regard to the determinants of antenatal depression, this study has found out that the factors significantly associated were marital conflict, pregnancy planning, and social support.

The existence of marital conflict was found to be a significant factor associated with antenatal depression. Those women who had marital conflict were about six times more likely to have antenatal depression as compared to women who had no marital conflict. This finding was consistent with previous studies [2, 4, 10, 13]. This might be illustrated in such a way that the physiological and psychological changes occurring during pregnancy might influence women to seek out close partner support without which it may increase the probability of antenatal depression. The increase in women's sexual problems during the early months of pregnancy might affect partnership characteristics, which in turn contributes to antenatal depression. A study conducted in Korea [39] showed that antenatal depression was associated with bad marital communication and marital dissatisfaction.

Those women who had planned their current pregnancy were 96% less likely to have antenatal depression as compared to those women who had no planned pregnancy. This finding was consistent with other studies [7, 30, 40, 41]. This is possibly because women who had planned pregnancy might be well prepared financially, psychologically, and socially for the phenomena of pregnancy and childbearing practice.

Women who had medium social support were 79% less likely to have antenatal depression as compared to those women who had low social support. Similar finding was reported from other studies [10, 18, 29–33]. This might be explained that social support from women's partner, family, and friends might help them confront stressful life events by receiving emotional, material, and informational supports during pregnancy.

This study might have the following limitations. Since the study was hospital based, pregnant women with depression, who do not seek antenatal care service at Dubti Hospital, would not be caught. Beck's Depression Inventory is a screening tool. Therefore, making a diagnosis of antenatal depression based on this scale without the gold standard psychiatric examination could be difficult. The objective of this study was also to assess prenatal depression rather than postnatal depression. Hence, follow-up study may come up with comprehensive pictures of the problem among both pregnant and postpartum women.

## 5. Conclusions

Nearly one in five pregnant women attending antenatal care at Dubti Hospital had antenatal depression. Marital conflict, pregnancy planning, and social support were found significantly associated with antenatal depression. Therefore, Dubti Hospital should strengthen its effort on prevention of unplanned pregnancy. In addition, healthcare workers at the antenatal care clinic have to deal with marital conflict and social support as part of their routine assessment to avoid the complications through early detection of antenatal depression.

## Figures and Tables

**Table 1:** Sociodemographic characteristics of pregnant women **(n=357)** attending antenatal care at Dubti Hospital, Northeast Ethiopia, 2016.

**Variables**	**Frequency (n)**	**Percent (**%**)**
**Maternal age (in year)**		
<20	39	10.9
20-24	110	30.8
25-29	128	35.9
30-34	47	13.2
>34	33	9.2
**Residence**		
Urban	302	84.6
Rural	55	15.4
**Educational status**		
No formal education	52	42.6
Formal education	205	57.4
**Current marital status**		
Unmarried*∗*	12	3.4
Married	345	96.6
**Religion**		
Muslim	245	68.6
Christian^†^	112	31.4
**Ethnicity**		
Afar	172	48.2
Tigre	66	18.4
Amhara	103	28.9
Other^*μ*^	16	4.5
**Occupation**		
Housewife	238	66.6
Employed (salary paid)^$^	92	25.8
Running personal business	27	7.6
**Average family monthly income (ETB)**		
<=500	39	10.9
>500	318	89.1

*∗*Single, divorced, and widowed. †Ethiopian orthodox, protestant, and catholic. $ Government and private employee. *μ* Oromo, Wolaita. ETB: Ethiopian birr.

**Table 2:** Obstetric characteristics of pregnant women attending antenatal care at Dubti Hospital, Northeast Ethiopia, 2016.

**Variables**	**Frequency (n)**	**Percent (**%**)**
**Gravidity (n=357)**		
Primigravida	136	38.1
Multigravida	221	61.9
**Parity (n=357)**		
0	136	38.1
1	65	18.2
>=2	156	43.7
**Number of children (n=357)**		
0	138	38.7
1	64	17.9
2	83	23.3
>=3	72	20.1
**History of abortion (n=357)**		
No	344	96.4
Yes	13	3.6
**History of still birth (n=357)**		
No	353	98.9
Yes	4	1.1
**Complication in previous pregnancy(n=221)** **∗**		
No	166	75.1
Yes	55	24.9
**ANC follow up in previous pregnancy (n=221)** **∗**		
No	98	44.3
Yes	123	55.7
**Trimester of current pregnancy (n=357)**		
First	85	23.8
Second	133	37.3
Third	139	38.9
**Current pregnancy planning (n=357)**		
No	73	20.4
Yes	284	79.6
**Current pregnancy complication (n=357)**		
No	348	97.5
Yes	9	2.5

ANC: antenatal care. **∗**At least one visit.

**Table 3:** Psychiatric history and psychosocial support of pregnant women **(n=357)** attending antenatal care at Dubti Hospital, Northeast Ethiopia, 2016.

**Variables**	**Frequency (n)**	**Percent (**%**)**
**History of depression in the woman**		
No	299	72.8
Yes	97	27.2
**Family history of depression**		
No	348	97.5
Yes	9	2.5
**Maternity social support**		
Low	91	25.5
Medium	228	63.9
High	38	10.6
**Marital conflict in the last 12 months**		
No	260	72.8
Yes	97	27.2

**Table 4:** Psychoactive substance use among pregnant women **(n=357) **attending antenatal care at Dubti Hospital, Northeast Ethiopia, 2016.

**Variables**	**Frequency (n)**	**Percent (**%**)**
**Ever drunk alcohol**		
No	312	87.4
Yes	45	12.6
**Drunk alcohol in the last 12 months**		
No	317	88.8
Yes	40	11.2
**Current drinkers**		
No	336	94.1
Yes	21	5.9
**Ever chewed Khat**		
No	347	97.2
Yes	10	2.8
**Chewed khat in the last 12 months**		
No	351	98.3
Yes	6	1.7

**Table 5:** Factors associated with antenatal depression among pregnant women attending antenatal care at Dubti Hospital, Northeast Ethiopia, 2016.

**Variables**	**Antenatal depression**		**COR(95% CI)**	**AOR (95% CI)**
	**Yes**	**No**		
**Maternal education**				
No formal education	15	137	0.35(0.2,0.65)^*∗*^	0.75(0.21,2.68)
Formal education	49	156	1	1
**Income (Birr)**				
<=500	13	26	2.6(1.3,5.4)^*∗*^	0.66(0.07,6.02)
>500	51	267	1	1
**Parity**				
0	26	110	1	1
1	18	47	1.78(0.79,5.13)	0.43(0.01,29.1)
>=2	20	136	0.69(0.21,3.74)	0.11(0.001,9.92)
**Complication in previous Pregnancy**				
No	25	141	1	1
Yes	15	40	2.12(1.02,4.4)*∗*	0.59(0.13, 2.78)
**Pregnancy planning**				
No	46	27	1	1
Yes	18	266	0.04(0.02,0.08)^*∗*^	0.04(0.01,0.11)^*∗*^
**Social support**				
Low	35	56	1	1
Medium	19	209	0.145(0.08,0.27)^*∗*^	0.21(0.07,0.66)^*∗*^
High	10	28	0.57(0.25,1.3)	1.45(0.33,6.35)
**Marital conflict in the last 12 months**				
No	23	237	1	1
Yes	41	56	7.54(4.2,13.6)^*∗*^	6.45(2.1,17.9)^*∗*^
**Previous history of depression**				
No	38	261	1	1
Yes	26	32	5.58(3.0,10.4)^*∗*^	2.47(0.68,8.9)
**Maternal age**				
<20	7	32	1.59(0.42,5.98)	1.48(0.06,35.13)
20-24	17	93	1.33(0.41,4.25)	0.23(0.02,2.74)
25-29	24	104	1.67(0.54,5.21)	1.99(0.32,12.33)
30-34	12	35	2.49(0.72,8.54)	4.73(0.75,29.91)
>34	4	29	1	1
**Previous number of children**				
0	33	105	1.57(0.27,8.32)	3.71(0.63,21.74)
1	8	56	0.71(0.25,6.35)	1.02(0.3,3.44)
2	11	72	0.76(0.12,3.61)	0.28(0.06,1.44)
>=3	12	60	1	1
**ANC follow up in previous pregnancy**†				
No	23	75	1	1
Yes	17	106	0.52(0.26,1.05)	0.84(0.49,2.4)
**Trimester of current pregnancy**				
First	11	74	0.6(0.3,1.3)	0.24(0.05,1.08)
Second	26	107	1.01(0.6,1.8)	0.84(0.27,2.68)
Third	27	112	1	1
**Family history of depression**				
No	61	287	1	1
Yes	3	6	2.35(0.57,9.67)	0.18(0.01,3.85)

*∗*Significant at *p* < 0.05. ANC: antenatal care. †At least one visit. CI: confidence interval. COR: crude odds ratio. AOR: adjusted odds ratio.

## Data Availability

The data used to support the findings of this study are available from the corresponding author upon request.

## Abbreviations

ANC: Antenatal Care
AOR: Adjusted Odds Ratio
CI: Confidence Interval
SD: Standard Deviation
ETB: Ethiopian Birr
WHO: World Health Organization
BDI: Beck's Depression Inventory
EPDS: Edinburgh Postnatal Depression Scale
PHQ: Patient Health Questionnaire.
