# Corrigendum to “Prevalence of Antenatal Depression and Associated Factors among Pregnant Women Attending Antenatal Care at Dubti Hospital: A Case of Pastoralist Region in Northeast Ethiopia”

**DOI:** 10.1155/2019/3921639

**Published:** 2019-03-04

**Authors:** Yihalem Abebe Belay, Nurilign Abebe Moges, Fetuma Feyera Hiksa, Kassahun Ketema Arado, Misgan Legesse Liben

**Affiliations:** ^1^Department of Public Health, College of Health Science, Debre Markos University, Debre Markos, Ethiopia; ^2^Department of Nursing, College of Health Science, Debre Markos University, Debre Markos, Ethiopia; ^3^Assistant Professor, Department of Public Health, Faculty of Health Sciences, Woldia University, Amhara, Ethiopia

In the article titled “Prevalence of Antenatal Depression and Associated Factors among Pregnant Women Attending Antenatal Care at Dubti Hospital: A Case of Pastoralist Region in Northeast Ethiopia” [1], Dr. Misgan Legesse Liben was missing from the authors' list. Dr. Misgan Legesse Liben assisted in designing the study, data analysis, and data interpretation. The corrected authors' list is shown above.

Also, the Authors' Contributions section should be updated as follows:

“Yihalem Abebe Belay conceived, designed the study, supervised the data collection, and performed the data analysis and interpretation. Nurilign Abebe Moges, Fetuma Feyera Hiksa, Kassahun Ketema Arado, and Misgan Legesse Liben assisted in designing the study, data analysis, and data interpretation. Yihalem Abebe Belay, Nurilign Abebe Moges, and Misgan Legesse Liben drafted the manuscript. All authors read and approved the final manuscript.” These corrections have been applied in place.
